# COVAX and equitable access to COVID-19 vaccines

**DOI:** 10.2471/BLT.21.287516

**Published:** 2022-03-25

**Authors:** Katelyn J Yoo, Akriti Mehta, Joshua Mak, David Bishai, Collins Chansa, Bryan Patenaude

**Affiliations:** a Health Nutrition and Population, World Bank, 701 18th St NW, Washington, DC 20006, United States of America (USA).; b Johns Hopkins Bloomberg School of Public Health, Baltimore, USA.

## Abstract

**Objective:**

To evaluate equity in the allocation and distribution of vaccines for coronavirus disease 2019 (COVID-19) to countries and territories participating in the COVID-19 Vaccines Global Access (COVAX) Facility.

**Methods:**

We used publicly available data on the numbers of COVAX vaccine doses allocated and distributed to 88 countries and territories qualifying for COVAX-sponsored vaccine doses and 60 countries self-financing their vaccine doses facilitated by COVAX. We conducted a benefit–incident analysis to examine the allocation and distribution of vaccines based on countries’ gross domestic product (GDP) per capita. We plotted cumulative country-level per capita allocation and distribution of COVID-19 vaccines from COVAX against the ranked per capita GDP of the countries and territories to generate a measure of the equity of COVAX benefits.

**Findings:**

By 23 January 2022 the COVAX Facility had allocated a total of 1 678 517 990 COVID-19 vaccine doses, of which 1 028 291 430 (61%) doses were distributed to 148 countries and territories. Taking account of COVAX subsidies, we found that countries and territories with low per capita GDP benefited more than higher-income countries in the numbers of vaccines. The benefits increased further when the analysis was adjusted by population age group (aged 65 years and older).

**Conclusion:**

The COVAX Facility is helping to balance global inequities in the allocation and distribution of COVID-19 vaccines. However, COVAX alone has not been enough to reverse the inequality of total COVID-19 vaccine distribution. Future studies could examine the equity of all COVID-19 vaccine allocation and distribution beyond the COVAX-facilitated vaccines.

## Résumé

**Objectif:**

Mesurer le niveau d'équité en matière de répartition et de distribution des vaccins contre la maladie à coronavirus 2019 (COVID-19) dans les pays participant au mécanisme COVAX (COVID-19 Vaccines Global Access).

**Méthodes:**

Nous avons utilisé les données accessibles au public sur le nombre de doses de vaccin COVAX allouées et distribuées à 88 pays et territoires ayant droit aux lots offerts par COVAX, ainsi qu'à 60 pays finançant eux-mêmes leurs doses avec l'aide de COVAX. Nous avons effectué une analyse d'incidence des bénéfices afin d'étudier la répartition et la distribution des vaccins en fonction du produit intérieur brut (PIB) par habitant. Enfin, nous avons représenté, sous forme de graphique, la répartition et la distribution cumulées des vaccins contre la COVID-19 provenant de COVAX pour chaque habitant à l'échelle nationale, puis les avons comparées au classement du PIB par habitant des pays et territoires concernés, en vue de mesurer le niveau d'équité des avantages COVAX.

**Résultats:**

Au 23 janvier 2022, le mécanisme COVAX avait attribué 1 678 517 990 doses de vaccin contre la COVID-19, dont 1 028 291 430 (61%) ont été distribuées à 148 pays et territoires. En tenant compte des subsides COVAX, nous avons découvert que, ’en termes de nombre de vaccins, les pays et territoires possédant un faible PIB par habitant en ont davantage bénéficié que les pays à revenu plus élevé. Ce bénéfice s'est accru lorsque nous avons paramétré l'analyse selon la catégorie d'âge de la population (65 ans et plus).

**Conclusion:**

Le mécanisme COVAX aide à atténuer les inégalités de répartition et de distribution des vaccins contre la COVID-19. Néanmoins, lui seul ne suffit pas à inverser la tendance à la disparité en matière de distribution de tels vaccins. Les futures études pourraient s'intéresser à l'équilibre de répartition et de distribution de tous les vaccins contre la COVID-19, et pas uniquement ceux fournis dans le cadre du mécanisme COVAX.

## Resumen

**Objetivo:**

Evaluar la equidad en la asignación y distribución de las vacunas para la enfermedad por coronavirus de 2019 (COVID-19) a los países y territorios que participan en el Mecanismo de acceso mundial a las vacunas contra la COVID-19 (Mecanismo COVAX).

**Métodos:**

Se utilizaron los datos disponibles públicamente sobre el número de dosis de vacunas del Mecanismo COVAX asignadas y distribuidas a 88 países y territorios que cumplían los requisitos para recibir las dosis de vacunas patrocinadas a través del Mecanismo COVAX y a 60 países que autofinanciaban sus dosis de vacunas con el apoyo del Mecanismo COVAX. Se realizó un análisis de beneficios e incidencias para revisar la asignación y distribución de las vacunas según el producto interno bruto (PIB) per cápita de los países. Se trazó la asignación y distribución acumulativa per cápita a nivel de país de las vacunas contra la COVID-19 del Mecanismo COVAX frente al PIB per cápita clasificado de los países y territorios para generar una medida sobre la equidad de los beneficios del Mecanismo COVAX.

**Resultados:**

A 23 de enero de 2022, el Mecanismo COVAX había asignado un total de 1 678 517 990 dosis de vacuna contra la COVID-19, de las que 1 028 291 430 (61 %) se distribuyeron a 148 países y territorios. Teniendo en cuenta las subvenciones del Mecanismo COVAX, se comprobó que los países y territorios con un PIB per cápita bajo se beneficiaron más que los países con mayores ingresos en lo que respecta al número de vacunas. Los beneficios aumentaron aún más cuando el análisis se ajustó por grupos de edad de la población (mayores de 65 años).

**Conclusión:**

El Mecanismo COVAX está ayudando a equilibrar las desigualdades a nivel mundial en la asignación y distribución de las vacunas contra la COVID-19. Sin embargo, el Mecanismo COVAX por sí solo no ha sido suficiente para revertir la desigualdad en la distribución total de las vacunas contra la COVID-19. Los estudios futuros podrían analizar la equidad de toda la asignación y distribución de las vacunas contra la COVID-19 además de las vacunas que se obtienen a través del Mecanismo COVAX.

## ملخص

الغرض تقييم التكافؤ في تخصيص وتوزيع لقاحات مرض فيروس كورونا 2019 (كوفيد 19) على البلدان والأقاليم المشاركة في مرفق الحصول العالمي على لقاحات كوفيد 19 (COVAX).

الطريقة قمنا باستخدام البيانات المتاحة للعامة بخصوص عدد جرعات لقاح COVAX المخصصة والموزعة على 88 دولة وإقليم مؤهل لجرعات اللقاح التي ترعاها COVAX، و60 دولة تدفع بنفسها مقابل جرعات اللقاح الخاصة التي تقدمها COVAX. لقد أجرينا تحليلًا للمزايا والحوادث للتحقق من تخصيص وتوزيع اللقاحات على أساس نصيب الفرد من الناتج المحلي الإجمالي (GDP) للدول. لقد خططنا التخصيص التراكمي لنصيب الفرد من لقاحات كوفيد 19 من COVAX وتوزيعه على مستوى الدولة، مقابل نصيب الفرد من الناتج المحلي الإجمالي المصنف للدول والأقاليم لوضع مقياس للتكافؤ في الحصول على مزايا COVAX.

النتائج بحلول يوم 23 يناير (كانون الثاني) 2022، خصص مرفق COVAX إجمالي 1678517990 جرعة من لقاح كوفيد 19، منها 1028291430 جرعة (61%) تم توزيعها على 148 دولة وإقليم. مع الأخذ في الاعتبار إعانات COVAX، وجدنا أن الدول والأقاليم ذات الناتج المحلي الإجمالي المنخفض للفرد، قد استفادت أكثر من الدول ذات الدخل المرتفع في أعداد اللقاحات. زادت المزايا بشكل أكبر عندما تم ضبط التحليل حسب الفئة العمرية للسكان (الذين تبلغ أعمارهم 65 عامًا فأكثر).

الاستنتاج يساعد مرفق COVAX في موازنة حالات عدم التكافؤ العالمية في تخصيص وتوزيع لقاحات كوفيد 19. ومع ذلك، فإن COVAX وحده لم يكن كافياً لعكس حالة عدم التكافؤ في التوزيع الإجمالي للقاح كوفيد 19. يمكن للدراسات المستقبلية أن تختبر التكافؤ في تخصيص وتوزيع لقاح كوفيد 19 بما يتجاوز اللقاحات التي تقدمها COVAX.

## 摘要

**目的:**

评估参与全球新冠肺炎疫苗实施计划 (COVAX) 的国家和地区分配和分发新冠肺炎 (COVID-19) 疫苗情况的平等性。

**方法:**

我们使用了关于分配和分发给 88 个有资格获得 COVAX 赞助的疫苗试剂的国家和地区以及 60 个获得 COVAX 帮助自筹疫苗试剂资金的国家和地区的公开可用数据。我们采取效益分析方法检查基于各国家人均国内生产总值 (GDP) 的疫苗分配和分发。我们将来自 COVAX 的 COVID-19 疫苗分配和分发的国内人均累积情况与国家和地区的人均 GDP 排名情况进行对比，以衡量 COVAX 福利的公平性。

**结果:**

截至 2022 年 1 月 23 日，COVAX 机构共分配了 1,678,517,990 剂新冠肺炎疫苗，其中 1,028,291,430 剂（61%）已分发到 148 个国家和地区。考虑到 COVAX 补助金，我们发现人均 GDP 较低的国家和地区在疫苗数量方面比高收入国家受益更多。当按人口年龄阶层（65 岁及以上）调整分析时，其受益程度进一步增大。

**结论:**

COVAX 机构有助于平衡全球新冠肺炎疫苗分配和分发的不平等情况。然而，仅 COVAX 并不足以扭转所有新冠肺炎疫苗分配情况的不平等性。还需要开展进一步的研究才能检查所有新冠肺炎疫苗（不仅仅是 COVAX 赞助的疫苗）分配和分发情况的公平性。

## Резюме

**Цель:**

Оценить справедливость выделения и распределения вакцин от коронавирусной инфекции 2019 года (COVID-19) в странах и территориях, участвующих в механизме глобального доступа к вакцинам против COVID-19 (механизм COVAX).

**Методы:**

Авторы использовали общедоступные данные о количестве доз вакцины COVAX, выделенных и распределенных в 88 странах и территориях, отвечающих требованиям для доз вакцины, спонсируемых COVAX, и 60 странах, самостоятельно финансирующих дозы вакцины при содействии COVAX. Авторы провели анализ пользы и случаев заболеваний, чтобы изучить выделение и распределение вакцин на основе валового внутреннего продукта (ВВП) стран на душу населения. Авторы нанесли на график кумулятивное выделение и распределение вакцин против COVID-19 на душу населения от COVAX на уровне страны в сравнении с ранжированным ВВП на душу населения в странах и территориях для расчета показателя справедливости преимуществ COVAX.

**Результаты:**

К 23 января 2022 г. механизм COVAX выделил в общей сложности 1 678 517 990 доз вакцины против COVID-19, из которых 1 028 291 430 (61%) доз были распределены в 148 странах и территориях. Принимая во внимание субсидии от COVAX, авторы обнаружили, что страны и территории с низким ВВП на душу населения получили большую пользу от количества вакцин, чем страны с более высоким уровнем дохода. Польза увеличилась еще больше, когда анализ был скорректирован по возрастной группе населения (в возрасте 65 лет и старше).

**Вывод:**

Механизм COVAX помогает сбалансировать глобальное неравенство в выделении и распределении вакцин против COVID-19. Однако одного COVAX было недостаточно, чтобы преодолеть существующее неравенство в общем распределении вакцины против COVID-19. В будущих исследованиях можно было бы изучить справедливость выделения и распределения всех вакцин против COVID-19, помимо вакцин, распределенных с помощью механизма COVAX.

## Introduction

Equitable vaccine distribution can be a major factor towards global control of the coronavirus disease 2019 (COVID-19) pandemic.^1^ The COVID-19 Vaccines Global Access (COVAX) Facility was created to facilitate vaccine distribution, although it is unknown whether investments in the initiative have yielded equitable benefits across countries.^2^ There have been increasing concerns about vaccine nationalism where wealthy nations acquire a disproportionate share of global COVID-19 vaccines.^3^ As of 24 January 2022, only 9.7% (about 63 million) of people in low-income countries have received at least one dose of COVID-19 vaccine.^4^

Established in June 2020, the COVAX Facility is a vaccine acquisition mechanism for countries and territories unable to bargain directly with manufacturers.^5^ Financing for participants is dependent on need. The 92 countries and territories with a gross national income per capita of less than 4000 United States dollars (US$) qualify for the COVAX advance market commitment and are allocated COVAX-funded vaccines to cover up to 20% of their populations.^1^ Advance market commitment funding comes from bilateral and multilateral development partners, private industry and individual philanthropists.^6^^,^^7^ Countries and territories who participate in COVAX but do not qualify for advance market commitment have to self-finance their COVID-19 vaccine purchases. However, depending on their financial commitment, these countries are guaranteed COVAX-approved vaccine doses for 10–50% of their populations.^8^

COVAX-secured dose allocation follows the World Health Organization’s (WHO) allocation framework for fair and equitable access to COVID-19 health products.^9^ This framework recommends that all countries must receive doses to vaccinate high-risk and vulnerable people before roll-out of the vaccination programmes to the rest of the population. Although this framework seeks to achieve fairness in access to COVID-19 vaccines among countries, some scholars argue that the initial 20% coverage requirement fails to account for vulnerabilities existing in poorer countries and countries with large outbreaks of COVID-19.^3^^,^^10^^–^^14^ Nonetheless, adherence to the framework’s recommendation can allow equal distribution of COVAX benefits among countries relative to their population sizes.^15^

To assess the extent to which COVAX has fulfilled its commitment, we evaluated equity in the allocation and distribution of COVAX-facilitated COVID-19 vaccines to countries and territories by income group and by proportion of older people. The cross-country analysis will add to the evidence on whether collaborative efforts such as the COVAX Facility can contribute to the equitable international allocation and distribution of scarce global public goods (in this case, vaccines) during international health emergencies. 

## Methods

### Data sources

We analysed secondary data on countries’ COVID-19 vaccine purchases, allocation and distribution, including data on the COVAX Facility and donations by bilateral and multilateral agencies, international nongovernmental organizations and private firms. We extracted the data from the United Nations Children’s Fund’s (UNICEF) COVID-19 vaccine dashboard as of 23 January 2022 at 20:00 Eastern Standard Time.^16^ We used the COVID-19 vaccine dashboard because it is the most comprehensive repository of up-to-date information on the distribution of the COVID-19 vaccines worldwide. Furthermore, UNICEF is leading efforts to procure and supply COVID-19 vaccines on behalf of the COVAX Facility.

To understand the differences between actual and intended distribution of COVAX benefits, we obtained: (i) allocated dose counts from the COVAX deliveries category of the UNICEF dashboard and (ii) distributed dose counts from the doses shipped subset of the COVAX allocation values. Vaccine allocation describes the projected number of COVAX vaccine doses available to the country, based on potential supplies and the allocation framework. The doses distributed describes the quantities of COVID-19 vaccines delivered to countries by COVAX at a given point in time.

Among the 148 countries and territories included on the dashboard, 88 countries qualified for COVAX-sponsored vaccine doses under the advance market commitment mechanism and 60 countries were self-financing their vaccine doses facilitated by COVAX. We grouped the countries into four income groups based on the World Bank country classification:^17^ 25 low-income countries (gross domestic product, GDP, per capita: less than US$ 1026), 55 lower-middle-income countries (GDP per capita: US$ 1026–3995), 43 upper-middle-income countries (GDP per capita: US$ 3996–12 375) and 25 high-income countries (GDP per capita: above US$ 12 375). Only three of the 92 countries and territories with advance market commitment were not included in the UNICEF dashboard: Burundi, Eritrea and Marshall Islands. Most upper-middle-income and high-income countries and territories had bilateral arrangements to obtain vaccines from other sources, which is not accounted for in this analysis. We used GDP per capita in US$ purchasing power parity (PPP) from the World Development Indicator database to rank countries and territories by income level. We used 2019 data which did not include the economic losses due to the COVID-19 pandemic. We obtained population data for 2020 from the United Nations Population Division. The focus of our study was equity across all COVAX participants. Other sources can shed light on vaccine allocations to crisis-affected populations.^18^ We only analysed cross-country and not intra-country allocation and distribution of COVID-19 vaccines.

### Data analysis

In line with COVAX guidelines and WHO’s fair allocation framework, we assumed that COVAX will fully subsidize vaccines for 20% of the population in countries and territories qualifying for advance market commitment.^9^ COVAX estimates state that the average cost per dose for those participating in COVAX is US$ 7.00 per dose for participants under the advance market commitment mechanism and US$ 10.55 per dose for countries and territories using self-financing.^19^ These costs include the costs of safety boxes and syringes (devices), UNICEF’s Supply Division procurement fees, freight and transport fees, and all other costs until arrival of the vaccines to the respective countries and territories. The estimate excludes cost categories such as labour and capital costs, cold chain and wastage or buffer stocks.

We used standard benefit–incident analysis methods for doses allocated and doses distributed to evaluate differences between actual and intended distribution of COVAX benefits. We performed the following steps: (i) ranking countries and territories from poorest to richest via per capita GDP adjusted for PPP; (ii) obtaining both COVAX vaccine doses allocated and distributed by country; (iii) estimating total per capita benefits received from COVAX; (iv) estimating self-financed per capita benefits that were facilitated by COVAX; (v) deducting self-financed per capita benefit from total per capita benefits to obtain COVAX-sponsored per capita benefits; and (vi) aggregating COVAX-sponsored per capita benefits. We plotted COVAX-sponsored per capita benefits on Lorenz concentration curves to assesses whether benefits were distributed equitably. A 45° line on the curves represents perfect equality and enabled us to quantify deviation from perfect equality. 

We then calculated Wagstaff concentration index (C):^20^
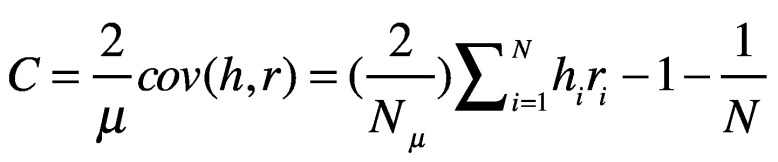
(1)where, *μ* is the average benefit from COVAX, and *cov(h,r)* is the weighted covariance between per capita COVAX benefit *h* received by country *i* and the country’s rank *r* in the GDP per capita distribution. The number of countries and territories, *N*, are ranked from 1 to *N*, that is, from poorest to richest. For computation, a more convenient formula for the concentration index defines it in terms of the covariance between the vaccine doses allocated or distributed and the fractional rank in the GDP per capita.^13^^,^^14^

When data are categorical rather than continuous, calculation of a standard concentration index may be insufficient. We therefore also calculated the Erreygers modified concentration index (MC), which accounts for the chosen transformation:^21^^,^^22^
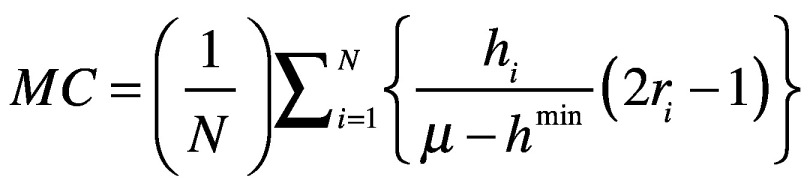
(2)where, *h^min^* is the lower limit of *h_i_*.

We analysed per capita COVAX benefits measured as country-level per capita COVAX expenditures on vaccines net of domestic expenditures on the vaccines, plotted against the ranked per capita GDP adjusted for PPP. We calculated Wagstaff and Erreygers concentration indices for total benefits, COVAX-sponsored benefits and self-financed per capita COVAX benefits for all countries and territories. We made the calculations for the total population of each country or territory. We also examined the distribution of COVAX benefits based on the proportion of the population aged 65 years and older as a proxy for the relative size of the most vulnerable population in each country or territory. 

For both indices, a concentration index of 0 to –1 reflects a pro-poor distribution, and an index of 0 to 1 reflects a pro-rich distribution.^23^ In a traditional benefit–incidence analysis approach, benefits are measured against individuals or entities ranked by an income metric. The analysis would therefore be based on individual-level data and the terms pro-rich or pro-poor would be used to refer to benefits accruing to different quintiles of the income distribution of countries. In our analysis we use the terms pro-rich to refer to COVAX benefits that were disproportionately accrued by wealthier countries and territories, as ranked by GDP per capita and adjusted for the size of the eligible population (and vice versa for the term pro-poor).

We used Excel (Microsoft Corp., Redmond, United States of America) and Stata version 16 (Stata Corp., College Station, USA) for the analysis.

## Results

### Vaccines allocated and distributed 

**Fig. 1:**
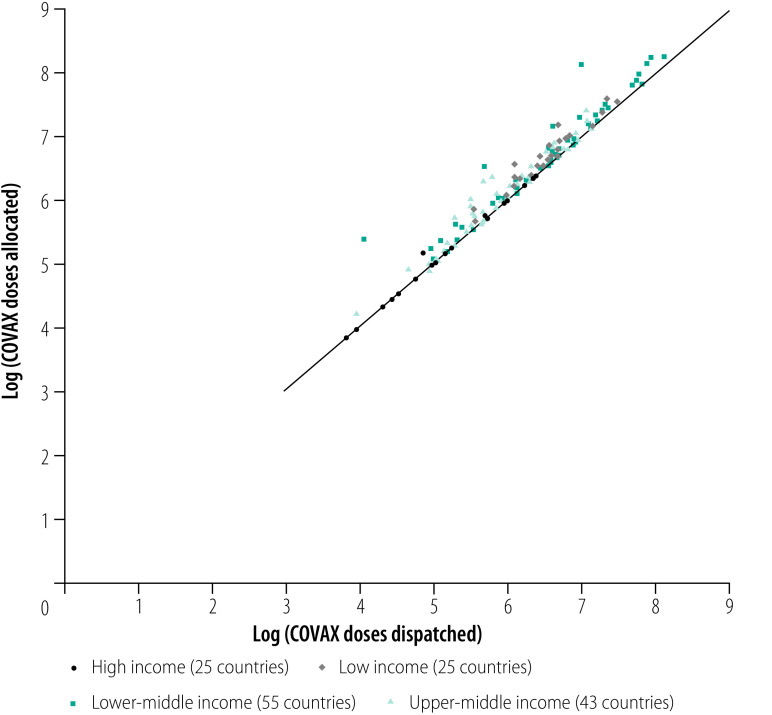
Log of vaccine doses allocated and distributed to 148 countries and territories participating in the COVAX Facility, by income levels, 23 January 2022

COVAX: COVID-19 Vaccines Global Access Facility; COVID-19: coronavirus disease 2019.

Note: Countries on the diagonal line have distributed all the vaccine doses allocated; those above the diagonal line still have unmet need.

**Fig. 2:**
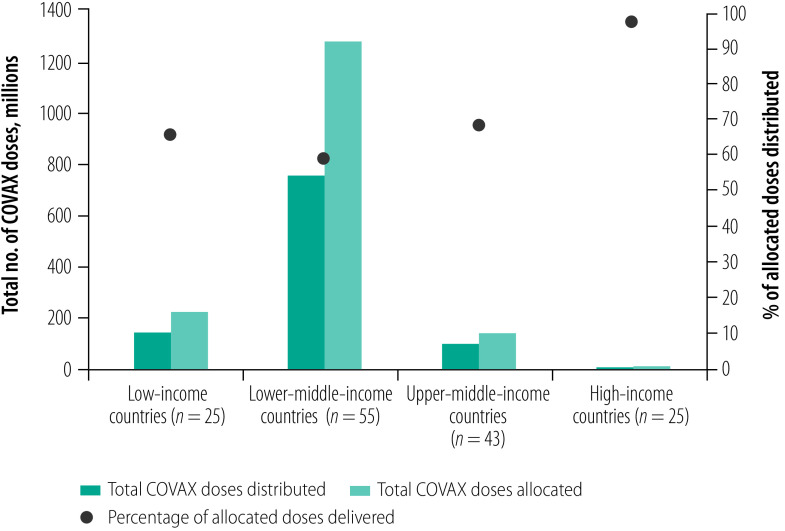
Total vaccine doses allocated and distributed to 148 countries and territories participating in the COVAX Facility, by income levels, 23 January 2022

COVAX: COVID-19 Vaccines Global Access Facility; COVID-19: coronavirus disease 2019.

Note: The average doses for each income group are not weighted for population sizes.

**Fig. 3:**
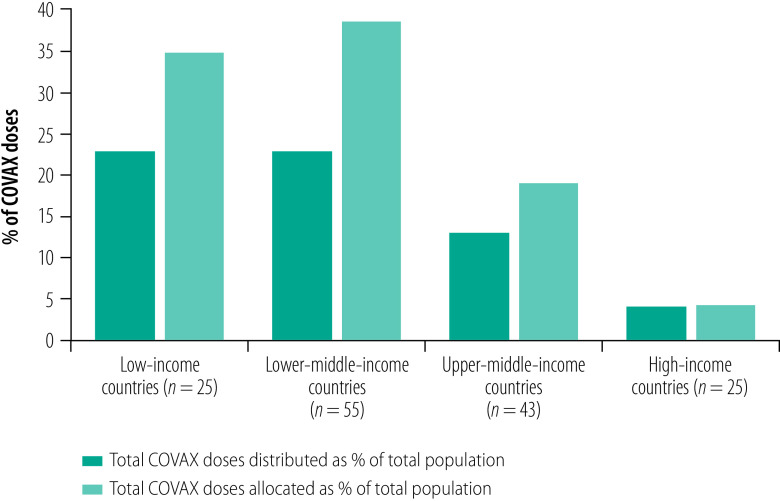
Total vaccine doses allocated and distributed to 148 countries and territories participating in the COVAX Facility, as a share of total population stratified by income levels, 23 January 2022

COVAX: COVID-19 Vaccines Global Access Facility; COVID-19: coronavirus disease 2019.

Note: The average doses for each income group are not weighted for population sizes.

At the time of analysis COVAX had allocated a total of 1 678 517 990 COVID-19 vaccine doses among 148 countries and territories, of which 1 028 291 430 (61%) doses had been distributed (Table 1). Fig. 1 demonstrates the disparity between the log of vaccine doses allocated and distributed to these countries and territories, while Fig. 2 and Fig. 3 stratify the doses by country income level as a share of the population. The lowest income group had relatively low total vaccine doses allocated and distributed in both absolute and relative terms to the population (Fig. 2). The lowest income group had one of the greatest gaps between the shares of total vaccine doses allocated and distributed to their populations (Fig. 3). Additional findings from the exploratory data analysis are presented in the authors’ online data repository.^24^

**Table 1:** Vaccine doses allocated and distributed to 148 countries and territories participating in the COVAX Facility, 23 January 2022

Country or territory, by log(y) COVAX doses allocated	Income group^a^	Advanced market commitment status	Total population 2020	GDP per capita PPP, current international $	No. of COVAX doses allocated	No. of COVAX doses distributed	Per capita no. of doses allocated	Per capita no. of doses distributed	Log(y) COVAX doses allocated	Log(x) COVAX doses distributed
Nauru	High income	Self-financing	10 834	14 099	7 200	7 200	0.66	0.66	3.86	3.86
Micronesia (Federated States of)	Lower-middle income	Sponsored	115 021	3 552	7 200	NA	0.06	NA	3.86	NA
Bermuda	High income	Self-financing	63 903	85 264	9 600	9 600	0.15	0.15	3.98	3.98
Tuvalu	Upper-middle income	Sponsored	11 792	4 456	16 800	9 600	1.42	0.81	4.23	3.98
Saint Kitts and Nevis	High income	Self-financing	53 192	27 345	21 600	21 600	0.41	0.41	4.33	4.33
Andorra	High income	Self-financing	77 265	49 900	28 740	28 740	0.37	0.37	4.46	4.46
Kuwait	High income	Self-financing	4 270 563	51 962	35 100	35 100	0.01	0.01	4.55	4.55
Antigua and Barbuda	High income	Self-financing	97 928	22 460	60 000	60 000	0.61	0.61	4.78	4.78
Tonga	Upper-middle income	Sponsored	105 697	6 648	81 800	91 800	0.77	0.87	4.91	4.96
Montenegro	Upper-middle income	Self-financing	621 718	23 344	84 000	48 000	0.14	0.08	4.92	4.68
New Zealand	High income	Self-financing	5 084 300	45 073	100 620	100 620	0.02	0.02	5.00	5.00
Brunei Darussalam	High income	Self-financing	437 483	64 724	100 800	100 800	0.23	0.23	5.00	5.00
Dominica	Upper-middle income	Sponsored	71 991	12 409	101 920	91 980	1.42	1.28	5.01	4.96
Bahrain	High income	Self-financing	1 701 583	46 966	107 820	107 820	0.06	0.06	5.03	5.03
Barbados	High income	Self-financing	287 371	16 300	114 840	114 840	0.40	0.40	5.06	5.06
Saint Vincent and the Grenadines	Upper-middle income	Sponsored	110 947	13 013	115 800	115 800	1.04	1.04	5.06	5.06
Kiribati	Lower-middle income	Sponsored	119 446	2 366	118 400	104 000	0.99	0.87	5.07	5.02
Qatar	High income	Self-financing	2 881 060	93 852	122 400	122 400	0.04	0.04	5.09	5.09
Grenada	Upper-middle income	Sponsored	112 519	17 771	124 710	114 630	1.11	1.02	5.10	5.06
Uruguay	High income	Self-financing	3 473 727	24 007	148 800	148 800	0.04	0.04	5.17	5.17
Seychelles	High income	Self-financing	98 462	28 685	154 440	74 880	1.57	0.76	5.19	4.87
Bahamas	High income	Self-financing	393 248	38 669	158 130	158 130	0.40	0.40	5.20	5.20
Belize	Lower-middle income	Self-financing	397 621	7 559	159 300	159 300	0.40	0.40	5.20	5.20
Suriname	Upper-middle income	Self-financing	586 634	19 842	165 600	144 000	0.28	0.25	5.22	5.16
Vanuatu	Lower-middle income	Sponsored	307 150	3 250	178 800	95 950	0.58	0.31	5.25	4.98
Trinidad and Tobago	High income	Self-financing	1 399 491	26 920	184 800	184 800	0.13	0.13	5.27	5.27
United Arab Emirates	High income	Self-financing	9 890 400	69 958	198 900	NA	0.02	NA	5.30	NA
Saint Lucia	Upper-middle income	Sponsored	183 629	16 102	202 470	197 430	1.10	1.08	5.31	5.30
Georgia	Upper-middle income	Self-financing	3 714 000	15 623	224 820	160 020	0.06	0.04	5.35	5.20
Sao Tome and Principe	Lower-middle income	Sponsored	219 161	4 175	237 120	129 120	1.08	0.59	5.37	5.11
Samoa	Lower-middle income	Sponsored	198 410	6 778	245 000	215 200	1.23	1.08	5.39	5.33
Comoros	Lower-middle income	Sponsored	869 595	3 189	250 380	12 000	0.29	0.01	5.40	4.08
Guyana	Upper-middle income	Sponsored	786 559	13 635	339 540	291 540	0.43	0.37	5.53	5.46
Cabo Verde	Lower-middle income	Sponsored	555 988	7 475	361 840	361 220	0.65	0.65	5.56	5.56
Djibouti	Lower-middle income	Sponsored	988 002	5 769	386 250	254 850	0.39	0.26	5.59	5.41
Albania	Upper-middle income	Self-financing	2 837 743	14 231	418 200	331 800	0.15	0.12	5.62	5.52
Solomon Islands	Lower-middle income	Sponsored	686 878	2 774	432 620	209 420	0.63	0.30	5.64	5.32
Eswatini	Lower-middle income	Sponsored	1 160 164	8 986	441 420	441 420	0.38	0.38	5.64	5.64
Dominican Republic	Upper-middle income	Self-financing	10 847 904	19 192	463 200	463 200	0.04	0.04	5.67	5.67
Gambia	Low income	Sponsored	2 416 664	2 317	477 420	376 800	0.20	0.16	5.68	5.58
Jordan	Upper-middle income	Self-financing	10 203 140	10 497	477 750	477 750	0.05	0.05	5.68	5.68
Bhutan	Lower-middle income	Sponsored	771 612	12 333	505 850	505 850	0.66	0.66	5.70	5.70
Fiji	Upper-middle income	Sponsored	896 444	14 263	500 800	501 280	0.56	0.56	5.70	5.70
Australia	High income	Self-financing	25 687 041	52 203	513 630	513 630	0.02	0.02	5.71	5.71
United Kingdom	High income	Self-financing	67 215 293	48 514	539 370	539 370	0.01	0.01	5.73	5.73
North Macedonia	Upper-middle income	Self-financing	2 083 380	17 583	552 420	201 420	0.27	0.10	5.74	5.30
Oman	High income	Self-financing	5 106 622	28 449	577 680	520 260	0.11	0.10	5.76	5.72
Maldives	Upper-middle income	Sponsored	540 542	20 357	581 770	371 170	1.08	0.69	5.76	5.57
Timor-Leste	Lower-middle income	Sponsored	1 318 442	3 703	587 640	393 420	0.45	0.30	5.77	5.59
Armenia	Upper-middle income	Self-financing	2 963 234	14 231	640 800	360 000	0.22	0.12	5.81	5.56
Mauritius	Upper-middle income	Self-financing	1 265 740	23 837	666 870	488 070	0.53	0.39	5.82	5.69
Gabon	Upper-middle income	Self-financing	2 225 728	15 582	688 830	472 200	0.31	0.21	5.84	5.67
Guinea-Bissau	Low income	Sponsored	1 967 998	2 021	763 200	360 000	0.39	0.18	5.88	5.56
Serbia	Upper-middle income	Self-financing	6 908 224	18 930	797 280	730 080	0.12	0.11	5.90	5.86
Bosnia and Herzegovina	Upper-middle income	Self-financing	3 280 815	15 847	835 740	332 640	0.25	0.10	5.92	5.52
Lesotho	Lower-middle income	Sponsored	2 142 252	2 693	917 490	653 670	0.43	0.31	5.96	5.82
Singapore	High income	Self-financing	5 685 807	102 573	938 400	938 400	0.17	0.17	5.97	5.97
Canada	High income	Self-financing	38 005 238	50 661	972 000	972 000	0.03	0.03	5.99	5.99
Taiwan, China	High income	Self-financing	23 871 085	24 502	1 020 000	1 020 000	0.04	0.04	6.01	6.01
Republic of Moldova	Upper-middle income	Sponsored	2 617 820	13 573	1 032 810	830 790	0.39	0.32	6.01	5.92
Namibia	Upper-middle income	Self-financing	2 540 916	10 262	1 055 980	332 640	0.42	0.13	6.02	5.52
Papua New Guinea	Lower-middle income	Sponsored	8 947 027	4 534	1 099 200	883 200	0.12	0.10	6.04	5.95
Haiti	Lower-middle income	Sponsored	11 402 533	3 028	1 124 700	805 480	0.10	0.07	6.05	5.91
Botswana	Upper-middle income	Self-financing	2 351 625	18 529	1 153 260	1 038 240	0.49	0.44	6.06	6.02
South Sudan	Low income	Sponsored	11 193 729	1 235	1 225 270	1 002 070	0.11	0.09	6.09	6.00
Mongolia	Lower-middle income	Sponsored	3 278 292	12 838	1 327 260	1 327 260	0.40	0.40	6.12	6.12
Kosovo^b^	Upper-middle income	Sponsored	1 775 378	11 972	1 325 190	739 620	0.75	0.42	6.12	5.87
Cameroon	Lower-middle income	Sponsored	26 545 864	3 796	1 521 850	1 380 750	0.06	0.05	6.18	6.14
Kyrgyzstan	Lower-middle income	Sponsored	6 591 600	5 481	1 528 800	1 428 000	0.23	0.22	6.18	6.15
Liberia	Low income	Sponsored	5 057 677	1 488	1 691 430	1 246 980	0.33	0.25	6.23	6.10
Jamaica	Upper-middle income	Self-financing	2 961 161	10 190	1 752 870	1 103 520	0.59	0.37	6.24	6.04
Saudi Arabia	High income	Self-financing	34 813 867	48 948	1 772 430	1 772 430	0.05	0.05	6.25	6.25
Malaysia	Upper-middle income	Self-financing	32 365 998	29 564	1 840 800	1 387 200	0.06	0.04	6.27	6.14
Azerbaijan	Upper-middle income	Self-financing	10 110 116	15 050	2 022 390	2 022 390	0.20	0.20	6.31	6.31
West Bank and Gaza Strip	Lower-middle income	Sponsored	4 803 269	6 510	2 097 560	1 362 620	0.44	0.28	6.32	6.13
Panama	Upper-middle income	Self-financing	4 314 768	32 761	2 074 350	484 320	0.48	0.11	6.32	5.69
Congo	Lower-middle income	Sponsored	5 518 092	4 005	2 124 850	1 882 710	0.39	0.34	6.33	6.27
Sierra Leone	Low income	Sponsored	7 976 985	1 793	2 258 910	1 510 110	0.28	0.19	6.35	6.18
Chile	High income	Self-financing	19 116 209	25 975	2 307 800	2 307 800	0.12	0.12	6.36	6.36
Costa Rica	Upper-middle income	Self-financing	5 094 114	21 792	2 359 860	648 150	0.46	0.13	6.37	5.81
Central African Republic	Low income	Sponsored	4 829 764	985	2 393 000	1 294 310	0.50	0.27	6.38	6.11
Paraguay	Upper-middle income	Self-financing	7 132 530	13 149	2 435 550	1 970 340	0.34	0.28	6.39	6.29
Republic of Korea	High income	Self-financing	51 780 579	42 728	2 516 580	2 516 580	0.05	0.05	6.40	6.40
Yemen	Low income	Sponsored	29 825 968	3 689	2 497 100	2 177 600	0.08	0.07	6.40	6.34
Lebanon	Upper-middle income	Self-financing	6 825 442	15 167	2 495 700	1 626 390	0.37	0.24	6.40	6.21
Benin	Lower-middle income	Sponsored	12 123 198	3 426	3 291 540	2 867 940	0.27	0.24	6.52	6.46
Mauritania	Lower-middle income	Sponsored	4 649 660	5 417	3 471 150	504 000	0.75	0.11	6.54	5.70
Mali	Low income	Sponsored	20 250 834	2 420	3 587 850	2 605 600	0.18	0.13	6.55	6.42
Madagascar	Low income	Sponsored	27 691 019	1 687	3 607 790	3 144 260	0.13	0.11	6.56	6.50
El Salvador	Lower-middle income	Sponsored	6 486 201	9 168	3 606 050	3 606 050	0.56	0.56	6.56	6.56
Libya	Upper-middle income	Self-financing	6 871 287	15 816	3 614 840	2 162 070	0.53	0.31	6.56	6.33
Chad	Low income	Sponsored	16 425 859	1 646	3 864 710	1 294 310	0.24	0.08	6.59	6.11
Cambodia	Lower-middle income	Sponsored	16 718 971	4 574	3 925 260	3 925 260	0.23	0.23	6.59	6.59
Zimbabwe	Lower-middle income	Sponsored	14 862 927	3 156	4 366 200	3 990 000	0.29	0.27	6.64	6.60
Togo	Low income	Sponsored	8 278 737	2 212	4 444 580	3 685 670	0.54	0.45	6.65	6.57
Malawi	Low income	Sponsored	19 129 955	1 579	5 014 350	2 813 850	0.26	0.15	6.70	6.45
Honduras	Lower-middle income	Sponsored	9 904 608	5 979	4 959 720	4 714 920	0.50	0.48	6.70	6.67
Niger	Low income	Sponsored	24 206 636	1 276	5 154 810	3 842 970	0.21	0.16	6.71	6.58
Sri Lanka	Lower-middle income	Sponsored	21 919 000	13 623	5 128 120	5 128 120	0.23	0.23	6.71	6.71
Guinea	Low income	Sponsored	13 132 792	2 676	5 270 480	4 793 310	0.40	0.36	6.72	6.68
Tunisia	Lower-middle income	Sponsored	11 818 618	11 210	5 426 350	4 519 020	0.46	0.38	6.73	6.66
Nicaragua	Lower-middle income	Sponsored	6 624 554	5 682	5 874 930	4 163 730	0.89	0.63	6.77	6.62
Ecuador	Upper-middle income	Self-financing	17 643 060	11 851	6 083 250	3 389 910	0.34	0.19	6.78	6.53
Somalia	Low income	Sponsored	15 893 219	903	6 434 930	5 096 900	0.40	0.32	6.81	6.71
Lao People's Democratic Republic	Lower-middle income	Sponsored	7 275 556	8 220	6 557 880	5 088 150	0.90	0.70	6.82	6.71
Mexico	Upper-middle income	Self-financing	128 932 753	20 448	6 563 940	6 563 940	0.05	0.05	6.82	6.82
Argentina	Upper-middle income	Self-financing	45 376 763	22 997	6 603 280	5 969 200	0.15	0.13	6.82	6.78
Senegal	Lower-middle income	Sponsored	16 743 930	3 504	6 973 520	3 770 990	0.42	0.23	6.84	6.58
Zambia	Lower-middle income	Sponsored	18 383 956	3 617	6 977 140	4 508 320	0.38	0.25	6.84	6.65
Guatemala	Upper-middle income	Self-financing	16 858 333	9 019	7 237 620	4 282 120	0.43	0.25	6.86	6.63
Burkina Faso	Low income	Sponsored	20 903 278	2 270	7 524 720	3 776 390	0.36	0.18	6.88	6.58
United Republic of Tanzania	Lower-middle income	Sponsored	59 734 213	2 773	7 522 380	7 522 380	0.13	0.13	6.88	6.88
Democratic People's Republic of Korea	Low income	Sponsored	25 778 815	1 700	8 115 600	NA	0.31	NA	6.91	NA
Ukraine	Lower-middle income	Sponsored	44 134 693	13 350	8 414 990	8 414 990	0.19	0.19	6.93	6.93
Peru	Upper-middle income	Self-financing	32 971 846	13 397	8 461 740	4 449 810	0.26	0.13	6.93	6.65
Democratic Republic of the Congo	Low income	Sponsored	89 561 404	1 144	8 901 400	5 149 740	0.10	0.06	6.95	6.71
Bolivia (Plurinational state of)	Lower-middle income	Sponsored	11 673 029	9 093	9 087 240	6 735 140	0.78	0.58	6.96	6.83
South Africa	Upper-middle income	Self-financing	59 308 690	13 010	9 269 910	9 269 910	0.16	0.16	6.97	6.97
Sudan	Low income	Sponsored	43 849 269	4 363	9 604 730	6 354 290	0.22	0.14	6.98	6.80
Tajikistan	Lower-middle income	Sponsored	9 537 642	3 733	9 614 360	8 069 720	1.01	0.85	6.98	6.91
Afghanistan	Low income	Sponsored	38 928 341	2 152	10 670 450	7 044 050	0.27	0.18	7.03	6.85
Iraq	Upper-middle income	Self-financing	40 222 503	11 012	11 898 780	8 598 750	0.30	0.21	7.08	6.93
Myanmar	Lower-middle income	Sponsored	54 409 794	5 297	12 252 600	NA	0.23	NA	7.09	NA
Brazil	Upper-middle income	Self-financing	212 559 409	15 388	13 881 600	13 881 600	0.07	0.07	7.14	7.14
Iran (Islamic Republic of)	Lower-middle income	Self-financing	83 992 953	12 913	14 423 650	13 115 310	0.17	0.16	7.16	7.12
Morocco	Lower-middle income	Sponsored	36 910 558	7 856	14 722 825	4 190 190	0.40	0.11	7.17	6.62
Rwanda	Low income	Sponsored	12 952 209	2 322	15 340 910	14 232 060	1.18	1.10	7.19	7.15
Syrian Arab Republic	Low income	Sponsored	17 500 657	4 685	15 587 640	4 892 840	0.89	0.28	7.19	6.69
Côte D’Ivoire	Lower-middle income	Sponsored	26 378 275	5 433	16 581 140	12 618 920	0.63	0.48	7.22	7.10
Ghana	Lower-middle income	Sponsored	31 072 945	5 625	18 478 400	16 616 490	0.59	0.53	7.27	7.22
Venezuela (Bolivarian Republic of)	Upper-middle income	Self-financing	28 435 943	17 528	18 584 400	12 076 800	0.65	0.42	7.27	7.08
Uzbekistan	Lower-middle income	Sponsored	34 232 050	7 311	21 041 690	9 855 590	0.61	0.29	7.32	6.99
Algeria	Lower-middle income	Sponsored	43 851 043	11 997	21 834 400	15 926 400	0.50	0.36	7.34	7.20
Mozambique	Low income	Sponsored	31 255 435	1 336	24 603 390	19 172 820	0.79	0.61	7.39	7.28
Kenya	Lower-middle income	Sponsored	53 771 300	4 513	26 746 470	19 401 270	0.50	0.36	7.43	7.29
Colombia	Upper-middle income	Self-financing	50 882 884	15 621	26 916 150	11 860 350	0.53	0.23	7.43	7.07
Nepal	Lower-middle income	Sponsored	29 136 808	4 120	30 406 390	22 926 920	1.04	0.79	7.48	7.36
Angola	Lower-middle income	Sponsored	32 866 268	6 952	32 323 830	21 564 180	0.98	0.66	7.51	7.33
Uganda	Low income	Sponsored	45 741 000	2 280	36 895 510	30 922 740	0.81	0.68	7.57	7.49
Ethiopia	Low income	Sponsored	114 963 583	2 315	40 813 010	22 461 170	0.36	0.20	7.61	7.35
Viet Nam	Lower-middle income	Sponsored	97 338 583	8 381	68 341 910	49 606 820	0.70	0.51	7.83	7.70
Philippines	Lower-middle income	Sponsored	109 581 085	9 292	69 869 275	65 724 200	0.64	0.60	7.84	7.82
Egypt	Lower-middle income	Sponsored	102 334 403	12 261	78 265 520	56 058 610	0.76	0.55	7.89	7.75
Nigeria	Lower-middle income	Sponsored	206 139 587	5 353	99 119 100	60 070 980	0.48	0.29	8.00	7.78
India	Lower-middle income	Sponsored	1 380 004 385	6 998	140 000 000	10 000 000	0.10	0.01	8.15	7.00
Pakistan	Lower-middle income	Sponsored	220 892 331	4 889	146 158 660	77 157 720	0.66	0.35	8.16	7.89
Indonesia	Lower-middle income	Sponsored	273 523 621	12 312	178 461 900	87 951 970	0.65	0.32	8.25	7.94
Bangladesh	Lower-middle income	Sponsored	164 689 383	4 955	192 439 610	133 062 580	1.17	0.81	8.28	8.12

COVAX: COVID-19 Vaccines Global Access Facility; COVID-19: coronavirus disease 2019; GDP: gross domestic product; NA: data not available; PPP: purchasing power parity.

^a^ Income groups are World Bank classifications.^17^

^b^ All references to Kosovo should be understood to be in the context of the United Nations Security Council resolution 1244 (1999).

Note: Countries are ordered from the lowest to highest log(y) COVAX doses allocated (Fig. 1).

### Benefit–incident analysis

#### Whole populations

**Fig. 4:**
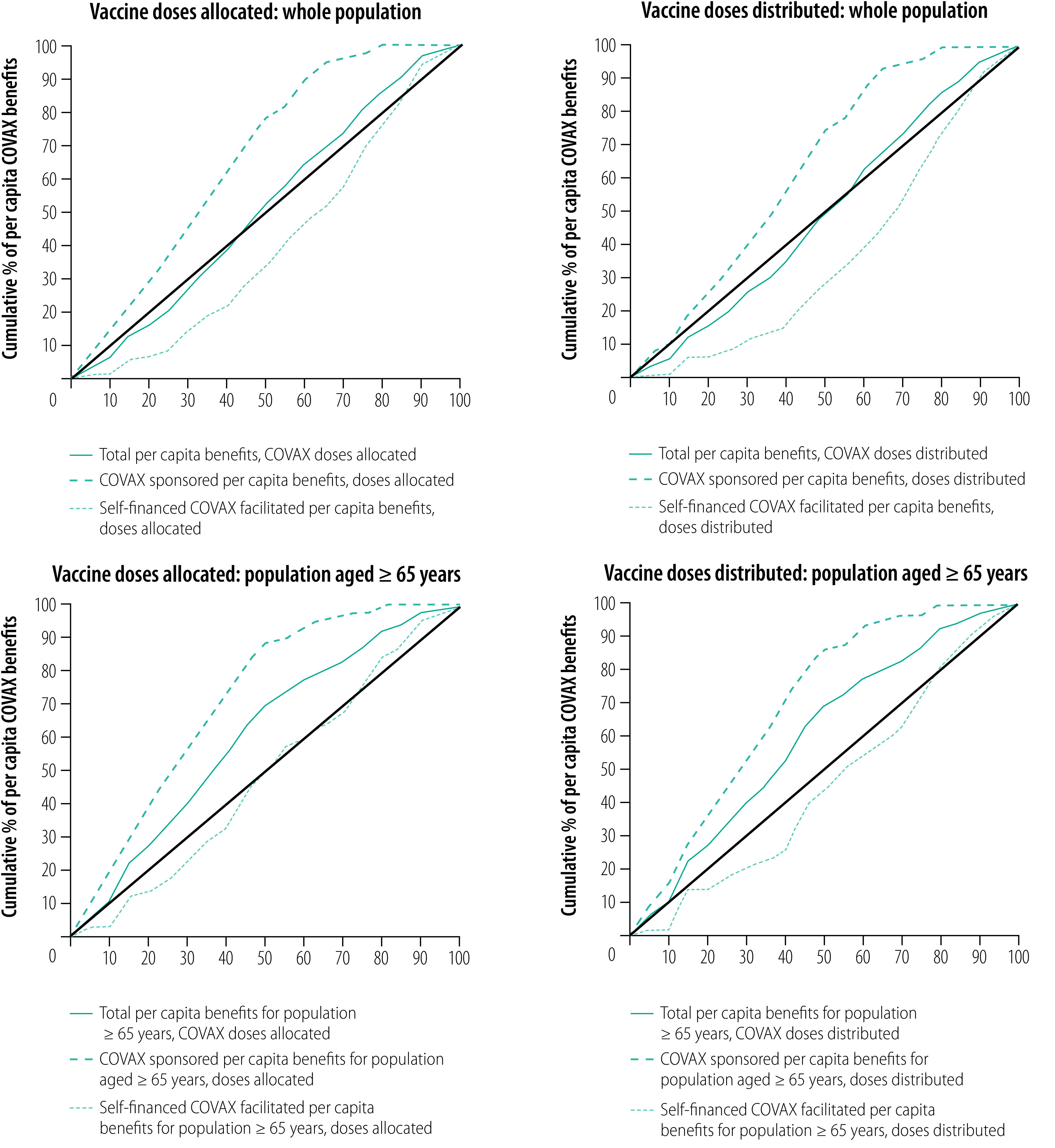
Concentration curves for per capita benefits accruing to 148 countries and territories participating in the COVAX Facility, 23 January 2022

COVAX: COVID-19 Vaccines Global Access Facility; COVID-19: coronavirus disease 2019.

Note: The black line depicts the line of perfect equality, whereby the poorest 10% countries (based on gross domestic product per capita adjusted for purchasing power parity) would receive 10% of the per capita COVAX benefits, poorest 20% countries would receive 20% of the benefits, and so on.

The concentration curve for total per capita COVAX benefits shows a pro-poor distribution, which lies mostly along the line of equality (Fig. 4). However, for the poorest 45% of countries and territories, a slight pro-rich trend is demonstrated. The concentration curve for the self-financed countries shows a disproportionate COVAX benefit to countries with higher per capita GDP but becoming slightly pro-poor for the wealthiest 15% of countries and territories. On the other hand, the concentration curve for the COVAX-sponsored per capita benefits consistently demonstrates pro-poor trends with about 50% of the poorest nations receiving about 80% of the benefits.

The average total per capita COVAX benefit was US$ 3.37 while the average COVAX-sponsored and self-financed per capita benefits were US$ 1.40 and US$ 1.98, respectively. The Wagstaff concentration indices for total per capita benefits and COVAX-sponsored per capita benefits were −0.034 and −0.657, respectively, indicating that the poorest 50% nations were allocated about 3% and 49% more total and COVAX-sponsored doses, respectively, compared with the wealthiest 50% nations, after adjusting for need (Table 2). In contrast, the index for self-financed per capita benefits (0.214) shows a disproportionate COVAX benefit to the least poor countries, indicating that about 16% of allocated doses would have to be transferred from the richest 50% countries to the poorest 50% countries to achieve need-based equity. The trend for Erreygers concentration indices was similar at −0.022 for total, −0.657 for COVAX-sponsored and 0.089 for self-financed COVAX-facilitated per capita benefits from doses allocated.

**Table 2:** Concentration indices showing per capita benefits accruing to 148 countries and territories participating in the COVAX Facility, 23 January 2022

Variable	Whole population					Population aged 65 years and older			
	Wagstaff concentration index	Relative dose benefit, %^a^	Erreygers concentration index	Relative dose benefit, %^b^		Wagstaff concentration index	Relative dose benefit, %^a^	Erreygers concentration index	Relative dose benefit, %^b^
**Vaccine doses allocated**									
Total benefits	−0.034	3	−0.022	2		−0.258	19	−0.176	13
Benefits to COVAX-sponsored countries	−0.657	49	−0.657	49		−0.577	43	−0.438	33
Benefits to self-financed COVAX-facilitated countries	0.214	16	0.089	7		0.057	4	0.031	2
**Vaccine doses distributed **									
Total benefits	−0.014	1	−0.012	1		−0.248	19	−0.159	12
Benefits to COVAX-sponsored countries	−0.518	39	−0.507	38		−0.514	39	−0.338	25
Benefits to self-financed COVAX-facilitated countries	0.298	22	0.164	12		0.120	9	0.054	4

COVAX: COVID-19 Vaccines Global Access Facility; COVID-19: coronavirus disease 2019.

^a^ Relative dose benefits calculated from Wagstaff concentration index.

^b^ Relative dose benefits calculated from Erreygers concentration index.

Note: We analysed data for a total of 148 countries and territories: 88 countries qualifying for COVAX-sponsored vaccine doses under the advance market commitment mechanism and 60 countries self-financing their vaccine doses facilitated by COVAX. We calculated both Wagstaff and Erreygers concentration indices as the Erreygers index accounts for when data are categorical rather than continuous.^22^ An index of 0 to –1 means that the benefits from COVID-19 vaccines supplied by the COVAX Facility are higher for countries and territories with low incomes based on GDP per capita adjusted for PPP (pro-poor). When the concentration index is positive, it signifies a relatively pro-rich distribution of benefits, while when the concentration index is negative, it implies a relatively pro-poor distribution. Relative dose benefit is computed from the formula (CI*75) to interpret the concentration index. This is the amount that would need to be linearly transferred from the top (bottom) 50% to the bottom (top) 50% of countries based on GDP per capita PPP to obtain perfect equality in benefits. For a concentration index which is positive, the relative dose benefit is the percentage of excess doses allocated or distributed to the richest 50% countries relative to the poorest 50% countries. For a concentration index that is negative, the relative dose benefit is the percentage of excess doses allocated or distributed to the poorest 50% countries relative to the richest 50% countries.

The concentration curves for per capita benefits from COVAX doses distributed mirror the trends seen in the curves for doses allocated (Fig. 4). The concentration curve for total per capita benefits lies along the line of equality and crosses it at the 49% mark, showing a disproportionate COVAX benefit to the poorest nations. A list of countries lying above and below the line of equality is shown in the author’s data repository.^24^ Self-financed per capita benefits were in favour of richer nations, although the curve crosses the line of equality at the 90% mark to become pro-poor. In contrast, the COVAX-sponsored per capita curve showed that benefits were consistently pro-poor, with about 50% of the poorest nations receiving about 75% of the benefits.

The average per capita benefits were US$ 2.46 for total, US$ 1.16 for COVAX-sponsored and US$ 1.29 for self-financed benefits. The Wagstaff concentration indices for total and COVAX-sponsored per capita benefits were pro-poor at −0.014 and –0.518, respectively (Table 2). Meanwhile, the index for self-financed per capita COVAX benefits at 0.298 favoured wealthier nations, indicating the need for a transfer of 22% of doses from the wealthiest 50% of the countries to the poorest 50% of the countries to achieve need-based equity. For reference, an index of –0.518 implies that the poorest 50% countries were receiving 39% more COVAX-sponsored doses than the richest 50% countries after adjusting for need, indicating that the financial benefits of COVAX are accruing to settings with lower ability to self-finance. Erreygers concentration indices for total (−0.012), COVAX-sponsored (−0.507) and self-financed COVAX-facilitated (0.164) per capita benefits showed similar findings to the Wagstaff concentration indices.

Benefits from allocated doses were more pro-poor compared with distributed doses. Additionally, the analysis demonstrates that self-financed expenditure both for doses allocated and doses distributed disproportionately benefited the richest nations in the absence of COVAX’s subsidies. When we took account of COVAX subsidies, we found that total and COVAX-sponsored per capita benefits were pro-poor for both allocated and distributed doses.

#### Vulnerable populations

The concentration curves and indices for COVAX-sponsored benefits adjusted for the size of the population aged 65 years and older are presented in Fig. 4 and Table 2. Similar to the whole population analysis, the concentration curves and indices for total and COVAX-sponsored per capita benefits were pro-poor, while the curves and indices for the self-financed COVAX-facilitated per capita benefits were in favour of wealthier nations, for both doses allocated and doses distributed. While the curves for whole population COVAX-sponsored benefits mirrored those after adjusting for the relative size of the older populations, the curves for total benefits adjusted for older populations were more pro-poor than the whole population benefits for both doses allocated and doses distributed. Compared with the whole population curves, the curve for self-financed benefits adjusted for older population size lay much closer to the line of equality for doses allocated, while the doses distributed still disproportionately benefited the wealthier nations, although less so. Overall, after accounting for the size of the population aged 65 years and older, there was an even greater pro-poor distribution of benefits compared with the overall population for both doses allocated and distributed. Additionally, the concentration indices for overall and adjusted for older populations showed that COVAX benefits were more pro-poor for allocated doses as compared with distributed doses.

## Discussion

We found that for both allocated and distributed COVID-19 vaccine doses, the total per capita benefits from the COVAX initiative disproportionately benefited countries and territories with lower per capita GDP. This difference applied when analysing the overall population and after accounting for the relative size of vulnerable older populations within each country. The total per capita benefits after adjusting for the size of older populations within each country demonstrated even higher benefits towards countries and territories with lower GDP per capita. These results were similar for COVAX-sponsored per capita benefits for both allocated and distributed COVID-19 vaccine doses.

The results also revealed that the benefits to poorer countries were greater for doses allocated than for doses distributed. This disparity can be explained by differences in the vaccine distribution systems across countries and territories. The differences include availability of cold-chain equipment, warehousing or storage capacities and human resources. Due to variations in supply-chain readiness, COVAX-eligible countries and territories may not receive their allocation from the COVAX Facility until minimum conditions are met. As such, WHO and UNICEF have developed a guidance note on COVID-19 vaccine supply and logistics management to help countries to prepare.^25^

We found variations in the benefits accrued across country income levels. Although total and COVAX-sponsored per capita benefits favoured poorer countries and territories, the benefits varied across country income levels, especially after adjusting for need using the size of the vulnerable older populations. In general, both total and COVAX-facilitated per capita benefits among self-financing countries disproportionately favoured countries with higher GDP per capita. This difference may be because nations with more resources can procure extra doses of COVID-19 vaccines in addition to the vaccines from the COVAX subsidy. These results also explain why the self-financed COVAX-facilitated per capita benefits accrued to nations with higher GDP per capita. However, the total benefits per capita favoured poorer countries when we took account of COVAX subsidies in the analysis. 

Despite substantial investments in vaccine delivery systems during the Global Vaccine Action Plan’s decade of vaccines (2010–2019), vaccine distribution systems of the poorest countries lag behind those of middle- and high-income countries.^26^^,^^27^ The performance gap may be partially due to previous vaccine investments focusing on reaching children, whereas addressing COVID-19 requires health systems to expand to reach the adult population. The ability to adapt to emerging challenges is a long-standing health-system goal that may have eluded past investments in vaccination systems in the poorest countries. Those countries who are facing discrepancies between the doses allocated and distributed may also face issues with allocating and distributing vaccines to the most vulnerable. Future progress on equity in the face of the current COVID-19 crisis will therefore require attention on the core capabilities of the health systems of the lowest income countries.

COVAX alone will not be sufficient to tackle future global inequity of vaccine access unless considerable reforms to the global system of vaccine governance are made. Although COVAX was able to allocate its COVID-19 vaccine doses among countries in an equitable manner, these efforts have not been enough to reverse the inequitable allocation and timely delivery of total COVID-19 vaccine. Inequities also still persist due to countries’ hoarding vaccine supplies for their own populations.^28^ The disparity in the total share of people vaccinated against COVID-19 between low-income and high-income countries remains large: more than 80% of the population in high-income nations compared with less than 10% of the population in low-income countries as of early 2022.^29^ This inequity in vaccine access exacerbates already overburdened health systems and economies and costs millions of lives globally, especially within lower-income countries. Without collective action from the international community and governments, paired with improvements in global vaccine equity mechanisms, the challenges will persist. 

There were some limitations to the study. First, we used PPP-adjusted GDP per capita to rank countries along a continuum. This country-level average does not reflect cross-country and in-country variations in living standards that may exist. Second, the analysis focused on the benefits received by countries and territories from COVAX in terms of the numbers of vaccine doses allocated and distributed. We were unable to determine how COVAX vaccine doses were allocated and distributed within the countries after the delivery by COVAX. Key issues such as human resources for health availability, geospatial access issues, internal stocking and cold-chain maintenance issues, and vaccine hesitancy may affect the ability of the countries and territories to eventually vaccinate their populations. As such, there may be significant variation in full vaccination coverage within and among the countries and territories. Another limitation is that we only examined doses from COVAX, omitting doses from other bilateral deals or non-COVAX sources. COVAX vaccines represent approximately 20% of all doses in circulation.^30^ Furthermore, our study was unable to assess the full effectiveness of COVAX, as we focused only on the allocation mechanism and not the procurement component. Lastly, the benefit–incident analysis assumes that expenditure on COVAX is an appropriate proxy for benefit. In reality, benefits are context-specific and require country-level epidemiological parameters to standardize the relative benefits of the additional doses across settings.

In conclusion, global risk-sharing for pooled procurement can foster the equitable distribution of COVID-19 vaccines and help to balance global inequities in the allocation and delivery of COVID-19 vaccines. Without COVAX subsidies and the COVAX Facility as a whole, poorer countries and territories may struggle to access COVID-19 vaccines. Therefore, expanding COVAX subsidies beyond 20% of the population for the poorer countries may be important to further enhance equity in the allocation and delivery of COVID-19 vaccines. Future studies could examine the equity of vaccine distribution within countries and include vaccines beyond the COVAX-facilitated vaccines.
